# Diastereoselective [2+2] Photocycloaddition of Chiral Cyclic Enones with Olefins in Aqueous Media Using Surfactants

**DOI:** 10.3390/molecules18021626

**Published:** 2013-01-28

**Authors:** Yasuhiro Nishiyama, Mikiko Shibata, Takuya Ishii, Tsumoru Morimoto, Hiroki Tanimoto, Ken Tsutsumi, Kiyomi Kakiuchi

**Affiliations:** 1 Graduate School of Materials Science, Nara Institute of Science and Technology (NAIST), 8916-5 Takayama-cho, Ikoma, Nara 630-0192, Japan; E-Mails: y-west@ms.naist.jp (Y.N.); s-mikiko@ms.naist.jp (M.S.); i-takuya@ms.naist.jp (T.I.); morimoto@ms.naist.jp (T.M.); tanimoto@ms.naist.jp (H.T.); 2 Department of Applied Chemistry, Faculty of Engineering, Miyazaki University, 1-1 Gakuen Kibanadai-Nishi, Miyazaki 889-2155, Japan

**Keywords:** diastereoselectivity, [2+2] photocycloaddition, aqueous media, surfactant

## Abstract

We conducted diastereodifferentiating [2+2] photocycloadditions of cyclo-hexenones modified with a chiral 8-(*p*-methoxy phenyl)menthyl auxiliary with olefins in water. Although the photoreaction didn’t proceed at all in pure water owing to very low solubility, the use of surfactants [sodium dodecyl sulfate (SDS) or dodecylamine hydrochloride (DAH)] and additive (organic solvent) enabled the reactions to progress with moderate to high conversions and yields. Furthermore, we synthesized a new menthol derivative substrate containing a (*p*-octyloxy)phenyl group for enhancing hydrophobicity, and elucidated that this new substrate was found to be a suitable chiral auxiliary in this asymmetric photoreaction in aqueous system. The additive effect of organic molecules on the yield and diastereoselectivity of the photo-adducts is also discussed.

## 1. Introduction

Recently within organic synthetic chemistry, environmentally friendly reaction systems have attracted a great deal of attention as “green chemistry” [1,2,3,4]. In these reactions, water is the most versatile reaction medium owing to its unique nature, nontoxicity, abundance, low cost and ease of waste treatment. Therefore, applications for organic reactions in water have been widely investigated aiming to eliminate both the use of volatile (and toxic, in some cases) organic solvents and the necessity of vigorous drying of solvents and substrates [5,6]. On the other hand, because many organic molecules are hydrophobic, we must modify the reaction systems to enhance solubility when trying reactions with organic molecules in water. 

Photochemical reactions have also been described as “green” chemical processes, because the driving force in reaction progress is simply light irradiation, which is considered clean energy [7,8]. Moreover, photoreactions have a complementary features compared to conventional thermal reactions. For example, in contrast with conventional thermal reactions, it is well known that photochemical [2+2] cycloaddition readily gives the distorted cyclobutane skeleton. Because this skeleton is synthetically versatile, not only for natural-product precursors such as terpenoids with polyquinane skeletons but also various unique compounds, much effort has been dedicated to this reaction, especially asymmetric [2+2] photoreaction [9,10]. However there are few reports concerned with the asymmetric photoreaction in aqueous media without use of a chiral host (such as a cyclodextrin). Previously, we examined the enantioselective [2+2] photocycloaddition of cyclohexenonecarboxylic acid with ethylene in water using cyclodextrin, and found the corresponding photocycloadducts were obtained in very low yield (~27%) and stereoselectivity (~5.5% enantiomeric excess) [11]. On the other hand, we have investigated a range of asymmetric [2+2] photocycloadditions in organic solvents and revealed that the diastereodifferentiating photoreaction using menthol derivatives as chiral auxiliaries is one of the most promising methods for achieving high diastereomeric excess (de) [12,13,14,15,16]. Thus, in this work, we focused on the diastereoselective [2+2] photocycloaddition of cyclohexenones having chiral menthol auxiliaries with olefins in water in order to develop a “greener” and more efficient asymmetric photoreaction process. In addition, in terms of solubility, a water-surfactant reaction system is better suited with organic reactions in aqueous media [17,18]. Thus we investigated the diastereoselective [2+2] photocycloaddition of cyclohexenones having chiral *p*-substituted 8-phenylmenthyl auxiliaries with olefins, ethylene or cyclopentene, in water with/without surfactants. In this study, we discuss the suitable menthol auxiliary and additive effect of organic molecules on yield and selectivity in both aqueous and organic reaction systems.

## 2. Results and Discussion

### 2.1. Synthesis of Substrates **3a**, **3b**

**Scheme 1:**
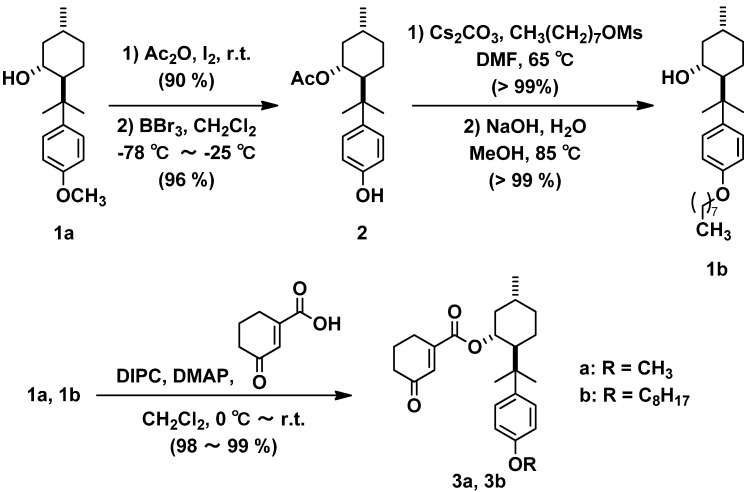
Synthetic route to substrates **3a**, **3b**.

Surfactants orient themselves in aqueous media to generate micelles, which due to the hydrophobic interaction include organic molecules inside. Thus we prepared (−)-8-[(*p*-octyloxy)phenyl]menthol (**1b**), which has a long alkyl chain to enhance hydrophobicity compared to that of (−)-8-[(*p*-methoxy)phenyl]menthol (**1a**) already reported (Scheme 1). For the assembly of a new-type chiral auxiliary, **1b** was prepared from (*R*)-(+)-pulegone according to our previous procedure modifying Corey’s method [12,13,14,19,20,21,14,19]. After the protection of **1a**, demethylation was carried out with borane tribromide to give the corresponding menthyl acetate **2** as reported previously [22]. The alkyl group was introduced into **2** and acetyl protection was removed to prepare the new menthol auxiliary **1b** almost quantitatively. The hydrophobic nature was increased by attaching an octyl unit. Therefore, we think that **1b** may be a more suitable chiral auxiliary than **1** for the reaction in an aqueous system using a surfactant. The condensation of cyclohexen-3-one-1-carboxylic acid with the menthols **1a**, **1b** with 1,3-diisopropylcarbodiimide (DIPC) and 4,4-dimethylaminopyridine (DMAP) gave chiral cyclohexenonecarboxylates **3a**, **3b** in high yields.

### 2.2. Photoreaction

#### 2.2.1. Photoreaction of **3a**, **3b** with Ethylene Gas as Coupling Partner

**Scheme 2:**
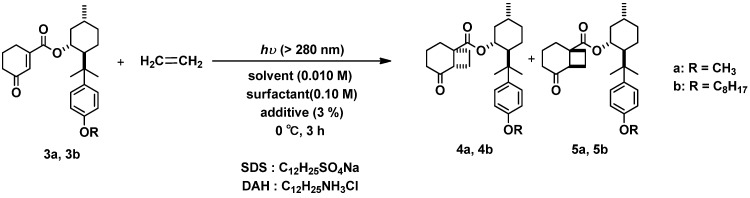
Diastereoselective [2+2] photocycloaddition of **3a**, **3b** with ethylene in aqueous media.

First of all, we tried the photoreaction of substrate **3a** with ethylene in pure water, however the reaction didn’t progress at all (Scheme 2, Table 1, entry 1). To drive this photoreaction in aqueous media, we added a surfactant [sodium dodecyl sulfate (SDS) or dodecylamine hydrochloride (DAH)] to this reaction. After bubbling the ethylene gas in water, SDS or DAH was added as a surfactant to the water solution followed by mixing with substrate **3a**. Then the photoreactions were carried out under similar conditions. Nevertheless, we could not observe the reaction proceeding (Table 1, entries 2 and 3). This could be explained by considering that substrate **3a** showed poor solubility in water, but its hydrophobicity was not large enough to be included in the micelle cavity. Then, we adopted compound **3b**, whose hydrophobicity was larger than that of **3a**, as a substrate and tried this photoreaction in aqueous media with/without surfactants. As anticipated, the reaction of **3b** in pure water did not progress owing to its large hydrophobicity (Table 1, entry 13). However contrary to our expectations, the reaction in aqueous media containing surfactants also didn’t progress at all (Table 1 entries 14–15). These results suggest that the photoreactions occurring in micelles produced with both surfactants and menthol derivatives **1a** or **1b** with ethylene gas were not suitable for proceeding in aqueous media.

**Table 1:** Diastereoselective [2+2] photocycloaddition of **3a**,**b** with ethylene *^a^*.

Entry	Substrate	Solvent	Surfactant	Additive	Conv./% *^b^*	Yield/% *^c^*	de/% *^b^*
						(4+5)	(4-5)/(4+5)
1	**3a**	H_2_O	−	−	0	*d*	*d*
2		H_2_O	SDS	−	0	*d*	*d*
3		H_2_O	DAH	−	0	*d*	*d*
4		H_2_O	SDS	CH_2_Cl_2_	100	83	23
5		H_2_O	SDS	toluene	100	73	32
6		H_2_O	SDS	MCH *^e^*	100	49	40
7		H_2_O	DAH	CH_2_Cl_2_	100	50	30
8		H_2_O	DAH	toluene	100	61	35
9		H_2_O	DAH	MCH *^e^*	100	41	40
10		CH_2_Cl_2_	−	−	100	83	19
11		toluene	−	−	100	104	31
12		MCH *^e^*	−	−	100	69	49
13	**3b**	H_2_O	−	−	0	*d*	*d*
14		H_2_O	SDS	−	0	*d*	*d*
15		H_2_O	DAH	−	0	*d*	*d*
16		H_2_O	SDS	CH_2_Cl_2_	62	52 *^f^*	52
17		H_2_O	SDS	toluene	73	16 *^f^*	42
18		H_2_O	SDS	MCH *^e^*	92	64 *^f^*	37
19		H_2_O	DAH	CH_2_Cl_2_	62	52 *^f^*	52
20		H_2_O	DAH	toluene	73	16 *^f^*	42
21		H_2_O	DAH	MCH *^e^*	92	64 *^f^*	37
22		CH_2_Cl_2_	−	−	100	98	12
23		toluene	−	−	100	99	23
24		MCH *^e^*	−	−	100	81	41

*^a^* Irradiated for 3 h at 0 °C. (See the Experimental Section.) *^b^* Determined by ^1^H-NMR. *^c^* Determined by the weight. *^d^* Not observed. *^e^* methylcyclohexane. *^f^* Isolated Yield.

Accordingly, we employed a new method of adding an organic solvent (3 volume %) into the aqueous media as an additive. The photoreaction of substrate **3a** or **3b**gave the corresponding photoadducts **4a** and **5a** or **4b** and **5b**, respectively, with moderate to high conversion (Table 1, entries 4–9, 16–21). It is clear that the additives affect the (**4**+**5**) reaction yield and diastereoselectivity (de: (**4**−**5**)/(**4**+**5**)). In the case of **3a**, the de values of photoproducts in aqueous media were mostly similar with those in organic solvent (Table 1 entries 4–9 *vs.* entries 10–12). It seems as if the reaction progressed in pure organic solvent, which was contained in the micelle. On the contrary, the de for the photoreaction of **3b** was highly enhanced by using additives compared to that in pure organic solvent, though we could not observe the full conversion (Table 1 entries 16–21 *vs.* entries 22–24). We previously reported that the de was affected by the relative population of the cyclohexenone-phenyl ring-stacking conformers of **3a** in the ground state. The **3a**’s conformation was stabilized by the electrostatic and/or dipole-dipole interactions between the C=O moiety of cyclohexenone and the OMe group attached to the aromatic ring [13,19]. In the confined hydrophobic reaction media in micelles, the hydrophobic bigger organic molecules may form the favorable conformer preferentially to afford the high selectivity. 

#### 2.2.2. Photoreaction of **3a,b** with Cyclopentene (liquid) as Coupling Partner

**Scheme 3:**
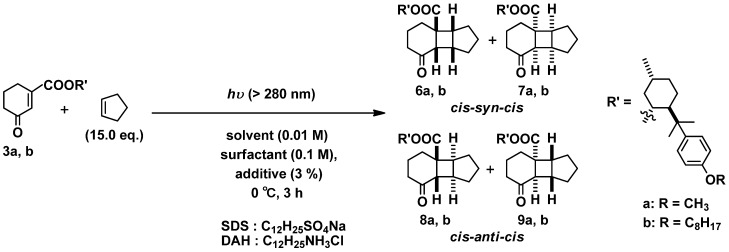
Diastereoselective [2+2] photocycloaddition of **3a**,**b** with cyclopentene in aqueous media.

In the former reaction, we could not achieve the full conversion photoreaction of **3b** with ethylene, even by using surfactants and additives, though we were able to enhance the diastereoselectivity compared the outcomes in pure organic solvent. In order to accomplish compatibility enhancing both reaction efficiency and stereoselectivity, we conducted the photoreaction with liquid olefin (cyclopentene) instead of gas olefin (ethylene) at 0 °C (Scheme 3). Though this photoreaction of **3a** should produce four diastereomeric isomers of photoadducts, *cis-syn-cis* products **6a**, **7a** and *cis-anti-cis* products **8a**, **9a** in organic solvents, only three products (**6**, **8**, and **9**) were observed as previous reported (Table 2, entries 10–12) [23]. The photoreaction of **3b** also gave only three products (compounds **6b**, **8b**, and **9b**) in organic solvent (Table 2, entries 22–24). The photoreactions of both **3a** and **3b** with cyclopentene in pure water did not progress at all. However, we could observe the reaction proceeding when adding the surfactant (SDS or DAH) without additive, which was different from the outcome in the case of using ethylene as coupling partner (Table 2, entries 1–3, 13–15). This can be explained by considering that cyclopentene was able to play the role not only as a coupling partner but also as an additive to solubilize the substrate in the micelle. 

The photoreaction of **3a** in H_2_O with surfactant gave the very low de compared to those in organic solvent (Table 2, entries 2, 3). As mentioned before, the de of this reaction of **3a** is sensitive to the solvent polarity. In aqueous media without additive, the high-polar water molecules may inhibit the interactions between the C=O moiety and OMe moiety to decrease selectivity. On the contrary, the de of this photoreaction of **3b** is not sensitive to the solvent polarity in the low polar solvent (Table 2 entries 22–24) Furthermore, both surfactants inhibited the de lowering, and achieved almost the same de as that in pure organic solvent (Table 2 entries 14, 15). Therefore, **3b**may be included in the micelle cavity mostly because of **3b**’s large hydrophobicity, and the photoreaction progressed in the low polarity circumstance inside the micelle cavity. 

**Table 2:** Diastereoselective [2+2] photocycloaddition of **3a**,**b** with cyclopentene*^a.^*

Entry	Substrate	Solvent	Surfactant	Additive	Conv./% *^b^*	Yield/% *^c^*	Ratio	de (%) *^d^*	
							(*syn*:*anti*) *^d^*		
						(6-9)	(6:(8+9))	syn 6	anti (8-9)/(8+9)
1	**3a**	H_2_O	−	−	0	*e*	*e*	*e*	*e*
2		H_2_O	SDS	−	100	87	39:61	>99	11
3		H_2_O	DAH	−	100	76	51:49	>99	11
4		H_2_O	SDS	CH_2_Cl_2_	100	66	56:44	>99	65
5		H_2_O	SDS	toluene	100	80	55:45	>99	60
6		H_2_O	SDS	MCH	100	63	59:41	>99	49
7		H_2_O	DAH	CH_2_Cl_2_	100	32	52:48	>99	53
8		H_2_O	DAH	toluene	100	40	49:51	>99	55
9		H_2_O	DAH	MCH	100	32	47:53	>99	54
10		CH_2_Cl_2_	−	−	100	88	53:47	>99	59
11		toluene	−	−	100	101	51:49	>99	86
12		MCH	−	−	100	83	52:48	>99	53
13	**3b**	H_2_O	−	−	0	*e*	*e*	*e*	*e*
14		H_2_O	SDS	−	100	47	43:57	>99	52
15		H_2_O	DAH	−	100	45	45:55	>99	54
16		H_2_O	SDS	CH_2_Cl_2_	100	28 *^f^*	43:57	>99	52
17		H_2_O	SDS	toluene	100	48 *^f^*	41:59	>99	53
18		H_2_O	SDS	MCH	100	66 *^f^*	43:57	>99	52
19		H_2_O	DAH	CH_2_Cl_2_	100	44 *^f^*	40:60	>99	43
20		H_2_O	DAH	toluene	100	49 *^f^*	40:60	>99	47
21		H_2_O	DAH	MCH	100	31 *^f^*	42:58	>99	37
22		CH_2_Cl_2_	−	−	100	77	40:60	>99	55
23		toluene	−	−	100	99	40:60	>99	53
24		MCH	−	−	100	81	40:60	>99	53

*^a^* Irradiated for 3 h at 0 °C. (See the Experimental Section.) *^b^* Determined by ^1^H-NMR. *^c^* Determined by the weight. *^d^* Determined by HPLC (CHIRALPAK AD). *^e^* Not observed. *^f^* Isolated Yield.

On the other hand, when using the additives, the de values of each substrate were quite different from the samples using ethylene gas. The de values of both substrates using SDS were almost the same as those in the organic solvent (Table 2 entries 4–6, 16–18). Cyclopentene can be miscible with both substrate and additive owing to cyclopentene’s nature, which is liquid and hydrophobic. This enables the full conversion of the reaction, especially in the case of **3b**. However, using DAH made the de values lower in both substrates (Table 2 entries 7–9, 19–21). In the anionic surfactant (DAH), the lower-polar additives than substrate might be included in the micelle cavity preferentially. Therefore, the substrate might be expelled from the cavity slightly, and the de lowering might be induced by the high-polar water.

## 3. Experimental

### 3.1. General

Most commercially available reagents were used without further purification. All reactions and manipulations of air- and moisture-sensitive compounds were carried out under an atmosphere of dry nitrogen using standard vacuum line techniques. Optical rotations ([α]) were determined on a JASCO DIP-1000 digital polarimeter using the sodium D line. Infrared spectra (IR) were obtained on a JASCO FT/IR-4200. High resolution mass spectra (HRMS) were obtained on a JEOL JMS-700 instrument. Flash column chromatography was performed with Merck silica gel 60N. Proton nuclear magnetic resonance spectra (^1^H-NMR) and carbon nuclear magnetic resonance spectra (^13^C-NMR) were obtained on a JEOL JNM-ECP500NK. NMR spectra were reported as chemical shift in ppm (δ), multiplicity (s = singlet, d = doublet, t = triplet, q = quartet, td = triplet of doublet, ddd = double double doublet, m = multiplet), coupling constant (Hz), and integration. ^1^H-NMR spectra were reported as chemical shifts in ppm base on the peak of tetramethylsilane (TMS) (δ = 0.0 ppm). ^13^C-NMR spectra were reported as chemical shifts in ppm based on the middle peak of CDCl_3_ (δ = 77.0 ppm).

### 3.2. Materials

#### 3.2.1. Preparation of (1*R*,2*S*,5*R*)-5-methyl-2-(2-(4-(octyloxy)phenyl)propan-2-yl)cyclohexanol (**1b**)

A mixture of **2** [22] (200 mg, 0.689 mmol), Cs_2_CO_3_ (898 mg, 2.76 mmol) and CH_3_(CH_2_)_7_OMs (287 mg, 1.38 mmol) in dimethylformamide (DMF) (7.0 ml) was stirred for 12 h at 65 °C under nitrogen atmosphere. The reaction mixture was cooled to room temperature and water added. The resulting mixture was extracted with ethyl acetate. The organic layer was washed with brine, and dried over MgSO_4_. The resulting material was purified by silica gel column chromatography (Hexane: AcOEt = 8:1; R_f_ = 0.40). A mixture of the purified product (277 mg, 0.689 mmol) and 1.0 M NaOH aq. (3.5 mL) in ethanol (30 mL, 0.02 M) was stirred for 8 h at 85 °C under nitrogen atmosphere. The reaction mixture was cooled to room temperature and the solvent was removed under a reduced pressure. The resulting mixture was extracted with CH_2_Cl_2_. The organic layer was washed with brine, and dried over MgSO_4_. The resulting material was purified by silica gel column chromatography (hexane:EtOAc = 8:1; R_f_ = 0.27), and the product **1b** was obtained (248 mg, 99%). Colorless oil, [α]_D_^24^ = −0.03, c = 0.06 in MeOH; ^1^H-NMR (CDCl_3_) δ: 7.29 (2H, d, *J* = 5.8 Hz), 6.84 (2H, d, *J* = 5.8 Hz), 3.91 (2H, t, *J* = 6.4 Hz), 3.51 (1H, td, *J* = 10.4, 4.1 Hz), 1.85–1.83 (2H, m), 1.78–1.72 (3H, m), 1.69–1.62 (2H, m), 1.47–1.26 (17H, m), 1.03 (1H, ddd, *J* = 25.2, 13.0, 3.2 Hz), 0.92–0.86 (8H, m); ^13^C-NMR (CDCl_3_) δ:157.11, 142.78, 126.68(2C), 114.27(2C), 72.83, 67.81, 54.05, 45.19, 38.99, 34.87, 31.77, 31.54, 31.41, 29.28, 29.19, 29.13, 26.39, 26.03, 23.98, 22.59, 21.94, 14.04; IR (neat) 3551, 3437, 2921, 1609, 1511, 1470, 1386, 1293, 1249, 1184, 1122, 1026, 964, 829 cm^−1^; HRMS(ESI) Calcd for C_24_H_40_O_2_Na (M+Na)^+^383.2926, Found 383.2925.

#### 3.2.2. Preparation of (1*R*,2*S*,5*R*)-5-methyl-2-(2-(4-(octyloxy)phenyl)propan-2-yl)cyclohexyl 3-oxocyclohex-1-enecarboxylate (**3b**)

A mixture of **1b** (248 mg, 0.689 mmol), DMAP (39.7 mg, 0.325 mmol), and cyclohexenone carboxylic acid (135 mg, 0.964 mmol) in CH_2_Cl_2_ (3.45 mL) was stirred and dropped DIPC (320 μL, 2.07 mmol) at 0 °C. The mixture was warmed to room temperature and stirred for 12 h. The resulting mixture was added into water and extracted with diethyl ether. The organic layer was washed with brine, and dried over MgSO_4_. The resulting material was purified by silica gel column chromatography (hexane:EtOAc = 8:1; R_f_ = 0.23), and the product **3b** was obtained (330 mg, 99%). Colorless oil, [α]_D_^2^^4^ = −0.03, c = 0.06 in MeOH; ^1^H-NMR (CDCl_3_) δ: 7.13–7.11 (2H, m), 6.72–6.71 (2H, m), 6.23 (1H, s), 4.98 (1H, td, *J* = 10.7, 4.7 Hz), 3.93–3.81 (2H, m), 2.35–2.31 (2H, m), 2.30–2.09 (2H, m), 2.08–2.00 (1H, m), 1.97–1.78 (4H, m), 1.76–1.67 (3H, m), 1.50–1.29 (10H, m), 1.28 (3H, s), 1.27–1.19 (2H, m), 1.16 (3H, s), 1.05–0.91 (2H, m), 0.91–0.86 (6H, m); ^13^C-NMR (CDCl_3_) δ:200.29, 165.25, 156.70, 148.65, 143.52, 132.32, 126.17(2C), 113.67(2C), 74.97, 67.68, 50.45, 41.77, 38.83, 37.51, 34.43, 31.83, 31.30, 29.64, 29.43(2C), 29.26, 26.33, 26.15, 24.37, 23.41, 22.67, 21.81, 21.78, 14.10; IR (neat) 2955, 1730, 1609, 1579, 1512, 1470, 1368, 1245, 1184, 1124, 1027, 982, 905, 829 cm^−1^; HRMS(ESI) Calcd for C_31_H_46_O_4_Na (M+Na)^+^ 505.3294, Found 505.3296.

#### 3.2.3. Photoproduct; (1*S*,6*S*)-(1*R*,2*S*,5*R*)-5-methyl-2-(2-(4-(octyloxy)phenyl)propan-2-yl)cyclohexyl 5-oxobicyclo[4.2.0]octane-1-carboxylate (**4b**, Major Compound)

Colorless oil, [α]_D_^2^^4^ = 1.2, c = 0.4 in MCH; ^1^H-NMR (CDCl_3_) δ: 7.17 (2H, d, *J* = 9.2 Hz), 6.79 (2H, d, *J* = 9.2 Hz), 4.90 (1H, td, *J* = 10.7, 4.3 Hz), 3.90 (2H, t, *J* = 6.7 Hz), 3.04 (1H, dd, *J* = 9.8, 7.6 Hz), 2.42–2.35 (1H, m), 2.30–2.18 (2H, m), 2.17 (1H, s), 2.14–2.06 (1H, m), 2.04–1.94 (2H, m), 1.94–1.88 (1H, m), 1.88–1.77 (4H, m), 1.77 (2H, d, *J* = 7.0 Hz), 1.70–1.62 (1H, m), 1.62–1.53 (2H, m), 1.50–1.40 (3H, m), 1.38–1.23 (11H, m), 1.19 (3H, s), 1.08–0.92 (2H, m), 0.88 (6H, m); ^13^C-NMR (CDCl_3_) δ:216.61, 175.08, 156.72, 143.08, 126.36(2C), 113.83(2C), 75.32, 67.85, 50.10, 48.47, 45.83, 41.75, 39.36, 38.91, 34.50, 31.83, 31.31, 30.92, 29.82(2C), 29.39, 28.26, 27.18, 26.94, 26.92, 26.10, 22.66, 21.79, 21.37, 20.75 14.11; IR (neat) 2927, 2869, 1709, 1250 cm^−1^; HRMS(ESI) Calcd for C_3__3_H_50_O_4_Na (M+Na)^+^ 533.3607, Found 505.3605.

#### 3.2.4. Photoproduct; (1*R*,6*R*)-(1*R*,2*S*,5*R*)-5-methyl-2-(2-(4-(octyloxy)phenyl)propan-2-yl)cyclohexyl 5-oxobicyclo[4.2.0]octane-1-carboxylate (**5b**, Minor Compound)

Colorless oil, [α]_D_^2^^4^ = −0.7, c = 1.1 in MCH; ^1^H-NMR (CDCl_3_) δ: 7.18 (2H, d, *J* = 8.5 Hz), 6.80 (2H, d, *J* = 8.5 Hz), 4.92 (1H, td, *J* = 10.7, 4.3 Hz), 3.92 (2H, t, *J* = 6.4 Hz), 2.93 (1H, t, *J* = 7.9Hz), 2.42–2.25 (2H, m), 2.21–2.12 (3H, m), 2.02–1.68 (10H, m), 1.63–1.57 (2H, m), 1.57–1.52 (2H, m), 1.48–1.39 (3H, m), 1.31 (6H, m), 1.29 (5H, m), 1.20 (3H, s), 0.89 (3H, d, *J* = 5.5 Hz), 0.87 (3H, t, *J* = 7.3 Hz); ^13^C-NMR (CDCl_3_) δ: 212.37, 175.28, 156.83, 142.97, 126.39(2C), 113.90(2C), 75.42, 67.89, 50.02, 48.46, 45.94, 41.87, 39.43, 38.89, 34.47, 31.84, 31.34, 30.37, 29.39(2C), 29.26, 28.23, 27.59, 27.01, 26.67, 26.12, 22.67, 21.79, 21.15, 20.89 14.12; IR (neat) 2952, 2856, 1714, 1250 cm^−1^; HRMS(ESI) Calcd for C_3__3_H_50_O_4_Na (M+Na)^+^ 533.3607, Found 533.3605.

#### 3.2.5. Photoproduct; (3a*R*,3b*S*,7a*S*,7b*S*)-(1*R*,2*S*,5*R*)-5-methyl-2-(2-(4-(octyloxy)phenyl)propan-2-yl)cyclohexyl 7-oxodecahydro-1H-cyclopenta[3,4]cyclobuta[1,2]benzene-3b-carboxylate (**6b**, *cis-syn-cis*, Major Compound)

Colorless oil, [α]_D_^2^^4^ = 2.0, c = 4.1 in MCH; ^1^H-NMR (CDCl_3_) δ: 7.16 (2H, d, *J* = 8.3 Hz), 6.77 (2H, d, *J* = 8.3 Hz), 4.95 (1H, td, *J* = 10.5, 4.4 Hz), 3.89 (2H, t, *J* = 6.4 Hz), 3.04 (1H, d, *J* = 6.1 Hz), 2.57 (1H, q, *J* = 7.9 Hz), 2.52–2.47 (1H, m), 2.29 (1H, td, *J* = 18.5, 5.3 Hz), 2.16–1.99 (3H, m), 1.95–1.83 (4H, m), 1.81–1.65 (4H, m), 1.55–1.39 (8H, m), 1.39–1.23 (9H, m), 1.18 (3H, s), 1.12–0.94 (4H, m), 0.93–0.80 (8H, m); ^13^C-NMR (CDCl_3_) δ:213.62, 175.56, 156.45, 142.46, 126.50(2C), 113.50(2C), 75.13, 67.99, 50.03, 48.13, 45.60, 45.46, 41.90, 39.16, 38.64, 34.50, 31.60, 31.52, 31.13, 30.99, 30.67, 29.39(2C), 29.26, 28.26, 27.61, 27.03, 26.45, 26.13, 22.57, 21.15, 21.12, 20.09, 14.13; IR (neat) 2927, 2869, 1708, 1137 cm^−1^; HRMS(ESI) Calcd for C_3__6_H_54_O_4_Na (M+Na)^+^ 573.3920, Found 573.3914.

#### 3.2.6. Photoproduct; (3a*S*,3b*S*,7a*S*,7b*R*)-(1*R*,2*S*,5*R*)-5-methyl-2-(2-(4-(octyloxy)phenyl)propan-2-yl)cyclohexyl 7-oxodecahydro-1H-cyclopenta[3,4]cyclobuta[1,2]benzene-3b-carboxylate (**8b**, *cis-anti-cis*, Major Compound)

Colorless oil, [α]_D_^2^^4^ = 0.8, c = 1.1 in MCH; ^1^H-NMR (CDCl_3_) δ: 7.14 (2H, d, *J* = 8.6 Hz), 6.80 (2H, d, *J* = 8.6 Hz), 4.82 (1H, td, *J* = 10.7, 4.3 Hz), 3.92 (2H, td, *J* = 6.6, 2.2 Hz), 3.01 (1H, d, *J* = 6.1 Hz), 2.70 (1H, q, *J* = 6.6 Hz), 2.56 (1H, t, *J* = 7.6 Hz), 2.45 (1H, td, *J* = 18.5, 5.3 Hz), 2.21–2.15 (1H, m), 2.11–2.01 (2H, m), 1.94–1.86 (2H, m), 1.79–1.72 (8H, m), 1.58–1.42 (7H, m), 1.39–1.25 (9H, m), 1.24 (3H, s), 1.17 (3H, s), 0.99–0.92 (2H, m), 0.89–0.84 (6H, m); ^13^C-NMR (CDCl_3_) δ:213.46, 175.46, 156.83, 142.97, 126.39(2C), 113.90(2C), 75.42, 67.89, 50.02, 48.46, 45.94, 45.46, 41.87, 39.43, 38.89, 34.47, 31.84, 31.80, 31.34, 30.81, 30.37, 29.39(2C), 29.26, 28.23, 27.59, 27.01, 26.67, 26.12, 22.67, 21.79, 21.15, 20.89, 14.12; IR (neat) 2928, 2871, 1708, 1187 cm^−1^; HRMS(ESI) Calcd for C_3__6_H_54_O_4_Na (M+Na)^+^ 573.3920, Found 573.3920.

#### 3.2.7. Photoproduct; (3a*R*,3b*R*,7a*R*,7b*S*)-(1*R*,2*S*,5*R*)-5-methyl-2-(2-(4-(octyloxy)phenyl)propan-2-yl)cyclohexyl 7-oxodecahydro-1H-cyclopenta[3,4]cyclobuta[1,2]benzene-3b-carboxylate (**9b**, *cis-anti-cis*, Minor Compound)

Colorless oil [α]_D_^2^^4^ = −1.1, c = 1.1 in MCH; ^1^H-NMR (CDCl_3_) δ: 7.15 (2H, d, *J* = 7.9 Hz), 6.77 (2H, d, *J* = 7.9 Hz), 4.89 (1H, td, *J* = 10.4, 4.4 Hz), 3.90 (2H, t, *J* = 6.4 Hz), 3.10 (1H, d, *J* = 6.1 Hz), 2.56 (1H, q, *J* = 7.9 Hz), 2.52–2.40 (1H, m), 2.29 (1H, m), 2.16–1.99 (3H, m), 1.95–1.83 (4H, m), 1.81–1.65 (4H, m), 1.55–1.43 (8H, m), 1.40–1.23 (9H, m), 1.19 (3H, s), 1.12–0.91 (4H, m), 0.83–0.73 (8H, m); ^13^C-NMR (CDCl_3_) δ:213.13, 175.76, 156.55, 142.43, 126.43(2C), 112.31(2C), 75.12, 67.73, 50.04, 48.30, 45.44, 45.42, 41.93, 39.06, 38.06, 34.54, 31.28, 31.50, 31.03, 30.79, 30.70, 29.43(2C), 29.36, 28.20, 27.46, 27.03, 27.00, 26.13, 22.51, 21.14, 21.02, 20.01, 14.03; IR (neat) 2928, 2829, 1702, 1127 cm^−1^; HRMS(ESI) Calcd for C_3__6_H_54_O_4_Na (M+Na)^+^ 573.3920, Found 573.3921.

### 3.3. Photolysis with Additive and Surfactant

#### 3.3.1. Photolysis of **3a,b** with Ethylene

Irradiation was carried out in a quartz cell placed in a Unisoku CoolspeK cryostat maintained at a given temperature, and then irradiated at wavelengths > 280 nm by a 500-W high-pressure mercury lamp through a 5-cm water layer and Pyrex glass filter, except for the reaction of **3b** with surfactant and additive. Irradiation of those samples was carried out in a Pyrex flask (>280 nm) installed in a water-cooled quartz immersion apparatus using HALOS 500 W high-pressure Hg lamp as the light source. A mixture of substrate, surfactants (0.10 M), additive (3%) and water (0.01 M) which was purged with ethylene for 10 min was irradiated at 0 °C. After 3 h irradiation, the resulting SDS mixture was extracted with diethyl ether. The organic layer was washed with brine, and dried over MgSO_4_ (In the case of using DAH, the resulting mixture was washed by NaHCO_3_ aq at first. Next, the mixture was extracted with diethyl ether after adding 2N-HCl aq. The organic layer was washed with brine, and dried over MgSO_4_.). After evaporation, each product (**4a**,**b**, **5a**,**b**) was observed as a mixture of diastereomers. Only for **3b** with surfactant and additive, we conducted column chromatography separation (hexane:EtOAc = 5:1 ; R_f_ = 0.37) for calculating yield because the separation was very difficult. The diastereomeric excess (de) value was determined by ^1^H NMR. The identification method was followed by analogous compounds in our previous work.

#### 3.3.2. Photolysis of **3a,b** with Cyclopentene

Irradiation was carried out in a quartz cell placed in a Unisoku CoolspeK cryostat maintained at a given temperature, and then irradiated at wavelengths >280 nm by a 500-W high-pressure mercury lamp through a 5-cm water layer and Pyrex glass filter, except for the reaction of **3b** with surfactant and additive. Irradiation of those samples was carried out in a Pyrex flask (>280 nm) installed in a water-cooled quartz immersion apparatus using HALOS 500 W high-pressure Hg lamp as the light source. A mixture of substrate, surfactant (0.10 M), additive (3%), and cyclopentene (0.075 M) and water (0.01 M) which was purged with nitrogen for 10 min were irradiated at 0 °C. After 3 h irradiation, the resulting SDS mixture was extracted with diethyl ether. The organic layer was washed with brine, and dried over MgSO_4_. (In the case of using DAH, the resulting mixture was washed by NaHCO_3_ aq. at first. Next, the mixture was extracted with diethyl ether after adding 2 N-HCl aq. The organic layer was washed with brine, and dried over MgSO_4_.) After evaporation, each photoadduct (**6a**, **b**, **8a**, **b**, **9a**, **b)** was obtained as diastereomers mixture. Only for **3b** with surfactant and additive, we conducted column chromatography separation (hexane:EtOAc = 5:1; R_f_ = 0.44 (**6b**), 0.37 (**8b**, **9b**)) for calculating yield because the separation was very difficult. The de value was determined by HPLC (CHIRALPAK AD, 1.0 mL/min, *n*-hexane/2-propanol = 98/2).

## 4. Conclusions

In summary, we developed the diastereoselective [2+2] photocycloaddition of chiral cyclohexenones with olefins in aqueous media. Contrary to our expectations, we could not make the photocycloadditions with ethylene gas proceed in water-surfactant (SDS) media, even after using a large hydrophobic substrate like **3b**. On the contrary, we could achieve the photoreactions using cyclopentene as a coupling partner in that media. By the addition of a small amount of CH_2_Cl_2_, toluene, or MCH, we succeeded in performing the asymmetric photoreactions in aqueous media similar to the reaction in organic solvents. Though we couldn’t observe a more efficient photoreaction using cyclopentene as coupling partner, we elucidated that the new-type chiral menthol auxiliary **1b** containing a (*p*-octyloxy)phenyl group was superior, especially in the asymmetric photoreaction using ethylene in aqueous solution. These results show a new and effective “green” reaction using light and water, with high yield and stereoselectivity.
